# Family socioeconomic position in early life and onset of depressive symptoms and depression: a prospective cohort study

**DOI:** 10.1007/s00127-016-1308-2

**Published:** 2016-11-11

**Authors:** Carol Joinson, Daphne Kounali, Glyn Lewis

**Affiliations:** 1 School of Social and Community Medicine, University of Bristol, Oakfield House, Oakfield Grove, Clifton, Bristol, BS8 2BN England, UK; 2 Division of Psychiatry, University College London, 67-73 Riding House St, London, W1W 7EJ England, UK

**Keywords:** Socioeconomic position, Depression, Depressive symptoms, Cohort study, ALSPAC

## Abstract

**Purpose:**

To investigate whether low parental socioeconomic position (SEP) at birth is associated only with early-onset depressive symptoms in offspring.

**Methods:**

This prospective cohort study used data on 9193 individuals (4768 females, 4425 males) from the Avon Longitudinal Study of Parents and Children. Depressive symptoms during three age periods (10–12, 12–16, 16–20 years) were assessed using the Short Mood and Feelings Questionnaire, and ICD-10 depression at age 18 was assessed using the Clinical Interview Schedule-Revised.

**Results:**

Low SEP was associated with increased incidence rates of depressive symptoms in all age periods, with indicators of low standard of living showing the strongest associations. For instance, incidence rate ratios for material hardship were 1.75 (95% CI [1.42–2.15]) at 10–12 years, 1.36 (1.16–1.61) at 12–16 years and 1.39 (1.21–1.59) at 16–20 years. Low SEP was also associated with increased odds of ICD-10 depression at 18 years, ranging from OR = 1.20 (95% CI [0.94–1.52]) for manual social class to 1.74 (1.35–2.24) for material hardship.

**Conclusions:**

There was no evidence that depressive symptoms can be “subtyped” by the age of onset, because the association with low SEP was evident for early- and later-onset symptoms. If socioeconomic inequalities in early life have long-term adverse impacts on mental health, policies addressing these inequalities could benefit the mental health of the population.

**Electronic supplementary material:**

The online version of this article (doi:10.1007/s00127-016-1308-2) contains supplementary material, which is available to authorized users.

## Introduction

It has been suggested that aetiological factors for depression differ according to whether onset occurred in childhood, adolescence or adulthood [1], and that risk factors in early life may only be associated with early-onset depression [2]. Jaffee and others [2] proposed distinct depression “subtypes” of juvenile-onset and adult-onset depression (17–25 years) and suggested that these are aetiologically distinct, though recognized that their results were based on small juvenile-depressed groups (comprising 21 individuals first diagnosed as having depression in childhood, but not in adulthood and 34 individuals first diagnosed in childhood whose depression recurred in adulthood by age 26 years). A ‘recency hypothesis’ was suggested by Shanahan and others [3] in which risk factors have time-limited depressogenic associations. They found, however, that early poverty was still associated with depression onset in young adulthood (19–21 years), albeit with a somewhat reduced strength of association. This study is, however, limited by relatively small numbers of new cases of depression and calculated odds ratios for depression in those exposed to risk factors compared with those who were never depressed during the entire follow-up period. This could lead to biased estimates where the length of follow-up differs among participants. It is better to use a method that incorporates time and calculates incidence rate ratios. Using such a method, Gilman and others [4] found that low socioeconomic position (SEP) did not differentiate among child-, adolescent-, and adult-onset depression. This study was, however, limited by retrospective assessment of age at depression onset.

There is evidence that exposure to low SEP in early life is associated with adverse developmental outcomes in children, including poor mental health [5]. SEP is a fundamental method of describing the structure of capitalist societies and low SEP is a marker of social adversity. Since certain dimensions of SEP (e.g. material living standards, educational attainment) can be altered by government policies, there is a potential route to prevention of mental health problems. It is unclear whether the association between early exposure to low SEP and mental health outcomes extends into adolescence and young adulthood. In this paper we use data from a large UK cohort to examine whether indicators of low parental SEP in early life are associated with an increased incidence of depressive symptoms between 10 and 20 years. We investigate whether low parental socioeconomic position (SEP) at birth is associated only with early-onset depressive symptoms in offspring. We examine whether there are differential associations of low SEP in early life on incidence of depressive symptoms in three age periods corresponding to late childhood (10–12 years), adolescence (12–16 years) and late adolescence/young adulthood (16–20 years). We also examine whether early exposure to low SEP is associated with increased odds of ICD-10 depression at 18 years.

## Method

### Participants

The sample comprised participants from the Avon Longitudinal Study of Parents and Children (ALSPAC). Detailed information about ALSPAC is available on the study website (http://www.bristol.ac.uk/alspac), which includes a fully searchable dictionary of available data (http://www.bris.ac.uk/alspac/researchers/data-access/data-dictionary). Pregnant women resident in the former Avon Health Authority in south-west England, having an estimated date of delivery between 1/4/91 and 31/12/92 were invited to take part, resulting in a cohort of 14,541 pregnancies and 13,973 singletons/twins (7217 boys and 6756 girls) alive at 12 months [6]. Ethical approval for the study was obtained from the ALSPAC Law and Ethics committee and local research ethics committees.

The sample used in the current study is based on the children from 15,247 pregnancies who were recruited in 1990–1992 along with the additional eligible cases who were recruited to the cohort at later phases. These pregnancies resulted in 15,458 fetuses, 14,775 were live births and 14,701 were alive at 12 months, of which 14,689 were singletons and twins. From these, we excluded 5496 who were missing all the outcome data in this study (depressive symptoms and depression) yielding a sample size of 9193.

### Measures

#### Depressive symptoms at 10–20 years

The Short Mood and Feelings Questionnaire (SMFQ) [7] enquires about the occurrence of depressive symptoms over the past 2 weeks. It correlates highly with the Children’s Depression Inventory (CDI) [8] and the Diagnostic Interview Schedule for Children (DISC) [9] and discriminates depressed from non-depressed children in general population samples [7].

The study children completed the SMFQ at six time points at mean ages: 10.6 years (SD = 0.26, range = 10–12.3 years); 12.8 years (SD = 0.23, range = 11.3–14.2 years), 13.8 years (SD = 0.21, range = 12.5–15.2 years), 16.7 years (SD = 0.24, range = 16.4–18.0 years); 17.8 years (SD = 0.42, range = 16.2–20.0 years) ;and 18.6 years (SD = 0.49, range = 17.8–20.1 years).

We dichotomized the SMFQ scale, defining high levels of depressive symptoms by scores at or above 11. This cutoff has been shown to have a high sensitivity and specificity [10] and has been applied in previous community samples [7, 10]. A cut point of 11 led to high sensitivity, specificity and negative predictive power for an ICD-10 diagnosis of depression at 18 years in the ALSPAC cohort [11].

#### Depression at 18 years

Participants also completed a self-administered computerized version of the Clinical Interview Schedule (CIS-R) [12] at a mean age of 17.8 years (SD = 0.42, range = 16.2–20.0 years). The CIS-R has been used extensively in earlier studies, e.g. in the UK Adult Psychiatric Morbidity Surveys of 1993, 2000 and 2007 and the 1958 Birth Cohort study. The CIS-R measures affective and anxiety disorders in the past week and enables diagnoses from the International Statistical Classification of Diseases, 10th Revision (ICD-10) for common mental disorders. The depression diagnosis examined in the current study is any depressive episode (mild, moderate or severe).

#### Indicators of SEP in early life

Three domains are often used to measure SEP: occupation, education and standard of living [13]. To assess SEP in these domains, we used data for the study from questionnaires completed by mothers around the time of the child’s birth. Occupational social class was assessed based on the lower of the mother or partner’s occupational social class using the 1991 British Office of Population and Census Statistics (OPCS) classification and dichotomized into non-manual [professional, managerial or skilled professions] and manual (partly or unskilled occupations).

Maternal educational attainment was defined as (1) A-level or above; (2) O-level; (3) certificate of secondary school education/vocational/none.

We used three binary indicators of standard of living: material hardship, home ownership (living in owner/occupier versus rented accommodation) and car access.

To complement these traditional measures of SEP, we also used a binary (yes/no) measure of perceived major financial problems reported by mothers. Correlations between these various SEP indicators were weak to modest.

### Analysis of the association between low SEP and onset of depressive symptoms

We derived empirical cumulative distributions for time to onset of depressive symptoms (defined as age in years and months at first occurrence of SMFQ ≥ 11) from non-parametric maximum likelihood estimates of the distribution function for interval-censored data using the EM algorithm in the statistical package R. Once an individual met criteria for a depressive episode, they were censored from any future analysis as they were no longer “at risk” of developing a first episode. We then used Poisson regression to model the age at onset of depressive symptoms as a function of gender and the SEP indicators. The Poisson model was equivalent to piecewise constant hazards specified for periods that correspond to stages associated with changes in the incidence of depressive symptoms: 10–12 years, i.e. 11 years 11 months; 12–16 years, i.e. 15 years 11 months and 16–20 years. This approach allows modelling of time-dependent associations. We fitted the models within the generalized linear modelling framework (GLM) using Stata 12.

Missing data patterns are summarized in online resource table S1. To deal with missing data, we used multiple imputations by fully conditional specification using chained equations (MICE) [14] in Stata 12. We conducted a sensitivity analysis to assess the direction of potential bias induced by MI assumptions by repeating the analysis, down-weighting observations more likely to be followed up. The imputation model included variables in addition to those included in this analyses that were either associated with missingness or were predictive of depression at age 18. These included maternal age, a range of indicators of family adversity and socio-demographics in pregnancy and early childhood (up to age 4). Please see online resource table S2 for full details of this weighting approach.

### Analysis for ICD10 depression at 18 years

We used logistic regression analysis to calculate the odds ratios for ICD10 depression according to our exposure variables.

## Results

### Association between gender and incidence of depressive symptoms

Table 1 displays the incidence rates of depressive symptoms by gender. The largest incidence rates for depressive symptoms occurred between 16 and 20 years.

**Table 1:** Age-specific incidence rates of depressive symptoms (SMFQ ≥ 11) per 100 person-years according to gender

Age group (years)	Person-years	Events (onset of depressive symptoms)	Rates^a^	95% CI^a^
Gender				
Male				
10–12 years	4782.39	219	4.58	[4.01, 5.23]
12–16 years	8312.99	265	3.19	[2.83, 3.59]
16–20 years	2719.99	399	14.67	[13.29, 16.18]
Female				
10–12 years	5279.99	232	4.39	[3.86, 4.99]
12–16 years	10,200	576	5.65	[5.20, 6.13]
16–20 years	3780.99	942	24.91	[23.37, 26.56]
Total	35,076.39	2633	7.51	[7.23, 7.79]

^a^Calculations are based on observed failure times (and ignore the fact that data are known in an interval and are not known exactly); confidence intervals are calculated using the quadratic approximation to the Poisson likelihood for the log-rate parameter

Table 2 shows the incidence rate ratios (IRRs) for the association of gender and depressive symptom onset. There was evidence for a time-dependent association of gender on depressive symptom incidence rates, with higher incidence rates in females compared with males at 12–16 and 16–20 years.

**Table 2:** Incidence rate ratios (IRR) for the univariate associations of each SEP indicator and gender with depression onset or with the timing/age of first occurrence of depressive symptoms

Characteristic	Units	Periods of onset time-dependent HR			Common HR
		<12 years HR 95% CI	(12–16) years HR 95% CI	>16 years HR 95% CI	(*F* test^(^*^)^, *p* value)^a^
Female	Binary				(*F*_2*df*_ = 10.19, *p* < 0.001)
		1.12 [0.93 1.35]	1.90 [1.65 2.19]	1.72 [1.53 1.92]	1.64 [1.52 1.78]
Manual social class	Binary				(*F*_2*df*_ = 1.63, *p* = 0.195)
		1.27 [1.04 1.54]	1.10 [0.95 1.28]	1.30 [1.16 1.46]	1.23 [1.13 1.33]
	Adjusted^(^**^)^	1.27 [1.04 1.54]	1.08 [0.93 1.25]	1.27 [1.13 1.42]	1.20 [1.11 1.31]
Rented accommodation	Binary	1.65 [1.32 2.05]	1.30 [1.09 1.56]	1.48 [1.28 1.71]	(*F*_2*df*_ = 1.34, *p* = 0.262)
					1.45 [1.31 1.61]
	Adjusted^(^**^)^	1.64 [1.31 2.05]	1.26 [1.05 1.51]	1.41 [1.22 1.63]	1.40 [1.27 1.55]
Major financial problems	Binary				(F_2*df*_ = 0.38, *p* = 0.686)
		1.48 [1.13 1.95]	1.61 [1.32 1.97]	1.44 [1.21 1.71]	1.51 [1.34 1.69]
	Adjusted^(^**^)^	1.48 [1.13 1.95]	1.61 [1.32 1.97]	1.42 [1.20 1.68]	1.49 [1.33 1.68]
No car access					(*F*_2*df*_ = 0.06, *p* = 0.939)
	Binary	1.54 [1.08 2.18]	1.49 [1.13 1.96]	1.59 [1.26 1.99]	1.54 [1.32 1.81]
	Adjusted^(^**^)^	1.53 [1.08 2.17]	1.44 [1.09 1.90]	1.53 [1.22 1.93]	1.50 [1.29 1.76]
Material hardship^b^	Binary				(*F*_2df_ = 2.01, *p* = 0.134)
		1.75 [1.42 2.15]	1.36 [1.16 1.61]	1.39 [1.21 1.59]	1.44 [1.32 1.59]
	Adjusted^(^**^)^	1.75 [1.42 2.15]	1.37 [1.16 1.61]	1.37 [1.20 1.57]	1.44 [1.31 1.58]
Maternal education	Categorical				(*F*_4df_ = 1.39, *p* = 0.236)
	≥A-level^c^	1	1	1	1
	O-level^d^	1.32 [1.04 1.65]	1.08 [0.92 1.27]	1.30 [1.15 1.47]	1.23 [1.12 1.34]
	CSE/vocational^e^	1.56 [1.22 1.99]	1.20 [0.99 1.45]	1.51 [1.30 1.74]	1.41 [1.27 1.56]
	Adjusted^(^**^)^				
	≥A-level	1	1	1	1
	O-level	1.31 [1.04 1.66]	1.07 [0.91 1.25]	1.26 [1.11 1.43]	120 [1.09 1.32]
	CSE/vocational	1.56 [1.22 1.99]	1.17 [0.97 1.41]	1.44 [1.23 1.66]	136 [1.23 1.51]

^a^Joint F test for the equality of regression coefficients across time periods averaged 100 imputations

^b^Material hardship was assessed using the question: “How difficult at the moment do you find it to afford these items? Food, clothing, heating, rent, items for child”: very difficult [score = 1], fairly difficult [score = 2], slightly difficult [score = 3] or not difficult [score = 4]. A score was calculated from these five variables using the algorithm: 20 minus the scores for each variable; thus scores ranged from 0 [lowest level of hardship] to 15 [highest]. A binary variable was derived using a cutoff of ≥5 corresponding to material hardship scores in the top 20% of the sample

^c^≥A-level [reference category]

^d^O-level

^e^CSE/vocational

(**): adjusted for gender

**Fig. 1:**
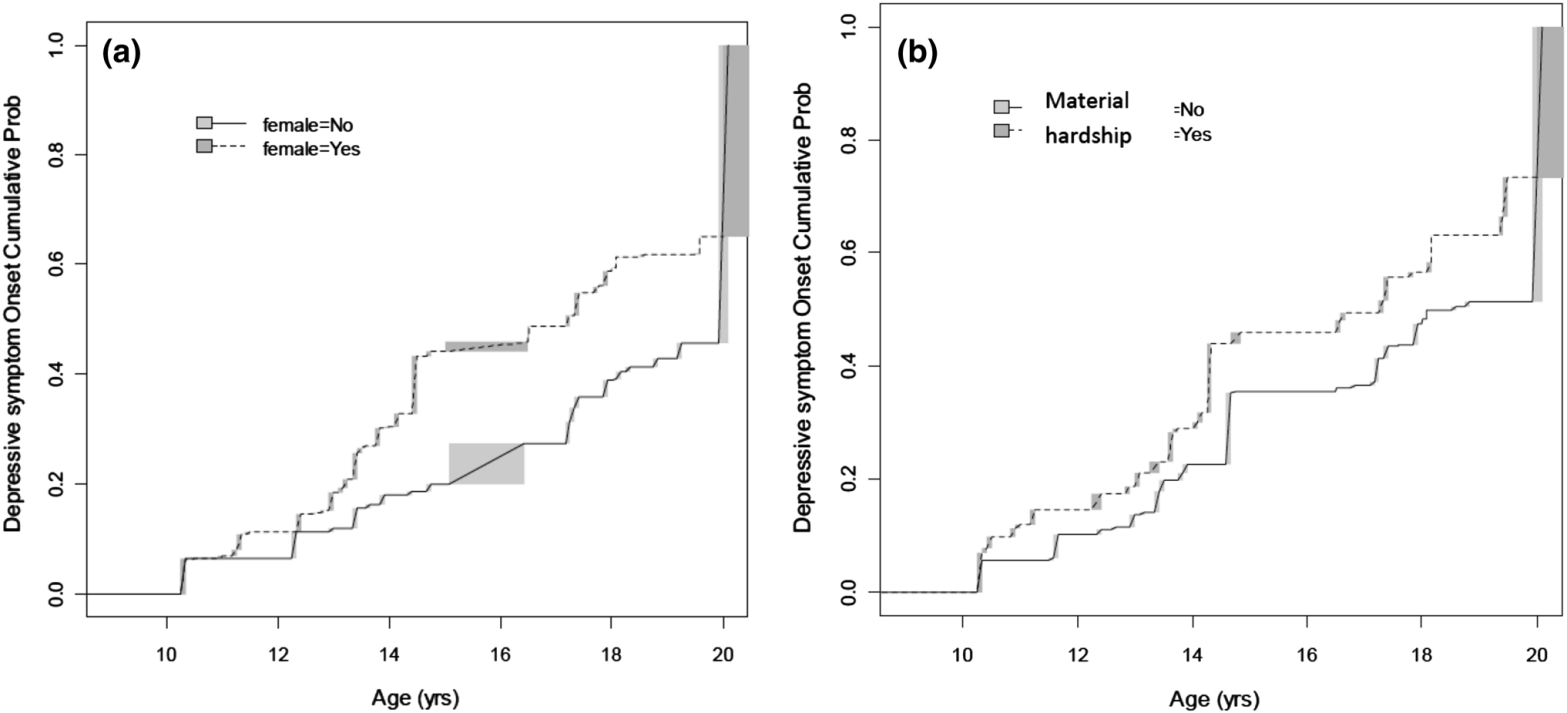
Cumulative probability of depressive symptom onset by **a** gender and age and **b** material hardship and age (changes in the nonparametric estimates of the survival function for interval-censored data usually do not occur at unique points, but occur within some interval—these intervals are shown as shaded areas on the figure)

Figure 1a shows the cumulative probability of onset of depressive symptoms in males versus females from age 10 to 20 years. There was an increasing probability of onset of depressive symptoms after 12 years in females compared with males.

We examined whether the apparent time-dependent association of gender on incidence of depressive symptoms could be due to differential loss to follow-up by conducting a sensitivity analysis to assess the direction of potential bias induced by the assumptions underlying multiple imputations by repeating the analysis, down-weighting observations more likely to be followed up. The results in table S2 indicate that this procedure reduced the apparent time dependency and females had higher depressive symptom incidence rates during all three age periods.

### Association between low SEP and incidence of depressive symptoms

Table 2 also shows the IRRs for the association of the SEP indicators with depressive symptom onset. There were associations between the SEP indicators and incidence of depressive symptoms, but there was no evidence that these were different across the three age periods.

When we repeated the analysis, down-weighting observations more likely to be followed up (table S2), the incidence rate ratios for all SEP indicators were less at 10–12 years compared with the results in Table 2. There was also greater uncertainty surrounding the estimates, suggesting no evidence for time dependencies.

We show the cumulative incidence of depressive symptoms by low SEP (using material hardship as an example) in Fig. 1b. This figure depicts the cumulative probability of age at onset of depressive symptoms for those born into families who experienced material hardship and those who did not.

We re-analysed our data using logistic regression (table S3) and found exaggerated time-dependent associations of exposure to low SEP on depressive symptoms in the early-onset group.

### Adjustments

We adjusted our results for gender, but estimates were unchanged. We also adjusted for antenatal maternal depression and single parenthood at birth and our results were robust (available on request). Such adjustments would be justified if we believed that these are causes of low SEP. Since it is unclear whether maternal depression and single parenthood are causes or consequences of low SEP, adjusting for these factors risks the introduction of bias [15].

### Predictors of ICD-10 depression at 18 years

Table 3 displays the odds ratios for the association of depression at age 18 with previous depressive symptoms and the SEP indicators. Those with depressive symptoms in childhood or adolescence had around a sevenfold increase in the odds of depression at 18 years. There was strong evidence that rented accommodation, major financial problems and material hardship were associated with increased odds of depression at age 18. In the mutually adjusted model (including all SEP indicators), material hardship was associated with increased odds of depression (OR = 1.57 [1.16, 2.12]), but odds ratios for the other SEP indicators were attenuated.

**Table 3:** Odds ratios [OR] for the univariable associations of ICD10 depression at 18 years with previous depressive symptoms and indicators of socioeconomic position

SEP indicator	*N*	OR 95% CI	OR 95% CI multiple imputations	OR 95% CI multiple imputations log[IMOR] = 1^a^	OR 95% CI multiple imputations mutually adjusted^b^
Previous depressive symptoms	4563	7.18 [5.58 9.24]	6.85 [5.20 9.05]	7.20 [5.60 9.26]	5.66 [4.19 7.64]
Manual social class	3984	1.20 [0.94 1.52]	1.16 [0.91 1.47]	1.47 [1.35 1.61]	0.99 [0.74 1.33]
Rented accommodation	4176	1.58 [1.20 2.09]	1.47 [1.11 1.95]	1.63 [1.45 1.82]	1.32 [0.94 1.83]
Major financial problems	3873	1.45 [1.04 2.01]	1.44 [1.04 1.99]	1.08 [0.95 1.24]	1.02 [0.70 1.49]
No car access	4173	1.54 [0.99 2.42]	1.36 [0.88 2.11]	1.60 [1.33 1.93]	0.97 [0.58 1.62]
Material hardship	4045	1.74 [1.35 2.24]	1.73 [1.34 2.24]	1.47 [1.32 1.63]	1.57 [1.16 2.12]
Maternal education	4152				
	≥ A-level	1	1	1	1
	O-level	1.27 [1.00 1.63]	1.24 [0.96 1.60]	1.12 [1.02 1.22]	1.20 [0.89 1.61]
	CSE/vocational	1.11 [0.82 1.52]	1.10 [0.80 1.53]	1.65 [1.49 1.84]	0.99 [0.68 1.47]

^a^The NMAR assumption is quantified through a missing not at random OR (IMOR). IMOR (informative missing OR) corresponds to the difference: delta = logit(Prob(case diagnosed if missing) − logit(Prob(case diagnosed if observed). IMOR of 0.1 would indicate a very strong association between being a ‘case’ (i.e. having depression) and missing data. A very small value of log(IMOR) close to 0 would represent an assumption of missing at random. The sensitivity analyses also assumed that missing data dependencies are distributed at random among those with low and high SEP

^b^Adjustment also included gender

## Discussion

We found evidence that early exposure to low SEP is associated with an increased incidence of depressive symptoms from 10 to 20 years. Depressive symptoms over this period were not “subtyped” by age of onset, because the association with low SEP was evident across this entire age range. The only time-dependent association was for the association between gender and SEP, with females having higher depressive symptom incidence rates at 12–16 and 16–20 years. Low SEP in early life was also associated with increased odds of ICD-10 depression at 18 years.

The strengths of our study include a longitudinal multi-wave design, large community-based sample, repeated measures of self-reported depressive symptoms and prospectively collected data on a range of SEP indicators. The availability of data spanning late childhood to late adolescence/young adulthood enabled us to examine how early exposure to low SEP affects the incidence of depressive symptoms over this entire age range. Having data on ICD-10 depression at 18 years allowed us to examine the association with early SEP at an age when depression rates approach those found in adults.

Calculation of incidence rate ratios, instead of odds ratios, is a major strength of our study, because this method reduced bias in the estimated associations between low SEP and depressive symptoms in the three age periods. Use of odds ratios could have led to biased estimates because of comparison of early incident cases with individuals who never became depressed during the whole follow-up period [3]. This might explain the stronger association of early risk factors on the onset of depression in childhood compared with adolescence and adulthood. When we re-analysed our data using logistic regression, we found exaggerated time-dependent associations of low SEP on depressive symptoms in the early-onset group (table S3). We also repeated the analysis using pubertal status at onset, rather than age, but results did not differ substantially (table S3). The first available self-reported measure of depressive symptoms in the ALSPAC cohort is the SMFQ at age 10. Cases of depressive symptoms that emerged before age 10 would, therefore, be included in the first age category. The incidence at 10–12 years might therefore be an overestimate.

Compared with earlier studies that used either a single measure of SEP (occupational social class) [2, 4] or a composite measure derived from a combination of indicators [3], we used a range of SEP indicators. It is recommended that studies examine a range of socioeconomic factors rather than limited aspects of SEP or composite measures [16]. This is because SEP is a complex and multidimensional social construct that is measured in epidemiological studies by a variety of indicators that often show only modest associations with each other and may tap into different causal pathways with different implications for policy [17]. For instance, low living standards may lead to parental stress and reduced capacity to invest resources in children, whilst low maternal education may result in poorer health literacy and decreased ability to communicate with, and access, health services. Consistent with an earlier study reporting that standard of living is more important for adult mental health than social class and education [13], we found evidence that indicators of standard of living (material hardship, home ownership and car access) had stronger associations with depressive symptoms and depression than either occupational social class or maternal educational attainment. It is notable that in the model adjusted for all SEP indicators, material hardship was strongly associated with increased odds of depression at age 18, whilst odds ratios for the other SEP indicators were attenuated. By creating a composite index we would not have been able to make these observations.

Assessment of SEP at birth is common in cohort studies examining the associations of early SEP on future health outcomes [18–20]. It is possible that children with low SEP at birth may no longer be classified as having low SEP at subsequent time points; however, this misclassification is likely to be non-differential with respect to our outcomes. We argue that SEP at birth will be an average indicator of SEP exposure across childhood. The finding of a sustained association between early SEP and onset of depressive symptoms up to the age of 20 years is striking, given that we have SEP measured only at birth.

Shanahan et al. [3] argued that childhood is a sensitive period for exposure to poverty, because the association they found between childhood poverty (assessed at age 9) and depression was not explained by poverty later in adolescence, using adjustment in a multivariable model. However, subsequent measures of poverty during adolescence could be on the causal pathway between childhood poverty and onset of depression. Bias can be induced by controlling for intermediate variables on the causal pathway between an exposure and outcome [15, 21].

Sample attrition in our study has implications for internal validity. Our study sample was socioeconomically advantaged compared with those who were lost to follow-up [6]. When we investigated the likely impact of missing data, we concluded that the complete case analyses [available on request] exaggerated time dependencies in the association between low SEP and depressive symptoms in childhood (10–12 years). After performing imputation of missing data and further sensitivity analyses, we found no evidence for time-dependent associations with low SEP. Attrition will also impact on the external validity of our study; therefore replication of our results in other samples with different distributions of socio-demographic variables would provide further reassurance of our findings.

Indicators of standard of living had stronger associations with depressive symptoms and depression than either occupational social class or maternal educational attainment. Although occupational social class and educational attainment are positively correlated with standard of living, social class is largely a measure of social status [22] and standard of living can vary at similar educational levels [16]. A poor material standard of living is characterized by economic and social deprivation, poor quality housing and neighbourhoods, and family pressures [23], and these factors affect the quality of the environmental exposures throughout development including cognitive stimulation, toxins, nutrition, parental stress and parent–child interactions [24]. Poverty is associated with adverse outcomes for early brain development [25] and mental health in children [26]. Links between low SEP, inconsistent parenting and less access to stimulating environments [27] could increase the risk of subsequent depression. By age 5, children from the poorest fifth of UK homes are, on average, a year behind in their development [28], which could set children on a trajectory for poor mental health [29]. Early adversity could establish a cognitive vulnerability in childhood that increases future susceptibility to depression when exposed to stressful life events [30]. Research is needed to identify the factors that might lie on the causal pathway between low SEP and depression.

### Policy implications

Our evidence for an association between early exposure to low SEP and onset of depressive symptoms from 10 to 20 years has important policy implications. Studies have reported a decline in social mobility in rich nations, including the UK and the USA, such that children who grow up in the poorest families are likely to remain in poverty as adults [31, 32]. The UK has recently seen a dramatic rise in socioeconomic inequality, with the gap between rich and poor growing faster than in any other rich country [33]. The number of children living in relative poverty is rising and it is estimated that by 2020 relative child poverty will be at its highest rate since 1999. The UK’s Child Poverty Act 2010 commits current and successive governments to eradicating child poverty by 2020, but recent findings suggest that current policies are falling short of government targets [34]. Children born into poverty are more likely to experience health and neurodevelopmental problems from birth and accumulate risks for poor outcomes throughout childhood [35]. We found that indicators of a poor material standard of living showed the strongest associations with depressive symptoms and depression. Material standard of living has the potential to improve through social and economic interventions [22]. Policies aimed at addressing socioeconomic inequalities, particularly improving living standards, in the early years could contribute to improving mental health in adolescents with consequent longer-term benefits for adults.

## Electronic supplementary material

Below is the link to the electronic supplementary material.

Supplementary material 1 (DOCX 72 kb)
