# Supplementary material for: Family socioeconomic position in early life and onset of depressive symptoms and depression: a prospective cohort study
Source: Soc Psychiatry Psychiatr Epidemiol. 2016 Nov 11;52(1):95–103. doi: 10.1007/s00127-016-1308-2 (PMC5226994; doi:10.1007/s00127-016-1308-2)
Supplement: Supplementary file 1 — Supplementary material 1 (DOCX 72 kb) [file 127_2016_1308_MOESM1_ESM.docx]

Electronic Supplementary Material

Article title: Family socioeconomic position in early life and onset of depressive symptoms and depression: a prospective cohort study

# Journal name: Social Psychiatry and Psychiatric Epidemiology

Authors: Carol Joinson^1^, Daphne Kounali^1^, Glyn Lewis^2^

Affiliations:

1. School of Social and Community Medicine, University of Bristol, Oakfield House, Oakfield Grove, Clifton, Bristol BS8 2BN.

2. Division of Psychiatry, University College London, 67-73 Riding House St London W1W 7EJ.

Corresponding author: Carol Joinson: [Carol.Joinson@bristol.ac.uk](mailto:Carol.Joinson@bristol.ac.uk), Phone: +44 117 331 3360.

Table S1: Counts of missing observations (%) for depression diagnosis and for each SEP indicator at 18 years in the whole sample as well as among those who developed any depressive symptoms during the whole assessment period

|  | **Missing counts** | |
| --- | --- | --- |
|  | **Sample size**  (N=9193)  N (%) | **Any Depressive symptoms present**  (N=2633)  N (%) |
| Depression at 18 years (CIS-R) | 4630 (50·4) | 1055 (40·1) |
| Social Class (manual) | 1334 (14·5) | 356 (13·5) |
| Rented Accommodation | 824 (8·9) | 236 (8·9) |
| Major Financial Problems | 1408 (15·3) | 393 (15·0) |
| No car access | 828 (9·0) | 234 (9·0) |
| Material hardship | 1138 (12·4) | 299 (11·4) |
| Maternal Education | 922 (10·0) | 240 (9·1) |

Missing depression data was strongly associated with gender, socioeconomic position and early loss of follow-up. Those with missing depression data were half as likely to have reported previous depressive symptoms (OR= 0.56, 95% CI= [0.51-0.61]). Heavy censoring on early depressive symptoms was strongly associated with missing data on depression at age 18. Girls were 30% less likely to have missing depression data (OR= 0.71 [0.65, 0.77]) and children from families of manual SES were 1.5 times more likely to be missing depression data (OR= 1.5 [1.3, 1.6]). Similar patterns of associations between missing depression data and the other SEP indicators were observed, with odds ratios for missingness as follows: rented accommodation: OR= 1.6 [1.4, 1.8]; no car access OR= 1.6 [1.3, 1.9]; material hardship OR= 1.4 [1.3, 1.6] and low maternal education OR= 2.1 [1.8, 2.3].

Table S2: Incidence rate ratios (IRR) for univariate associations of each SEP indicator and gender with depressive symptom onset (weighted with weights proportional to the probability of early drop-out across 100 imputations).

| **Characteristic** | **Units (F-test^1^, p-value)** | **Periods of Onset Time dependent IRR (IRR 95% CI)** | | | **Common IRR** |
| --- | --- | --- | --- | --- | --- |
|  |  | 10 – 12 years | 12 – 16 years | 16 – 20 years |  |
| Gender (female) | F_2df_=2·45,p=0·09 | 1·91 [1·59 2·29] | 1.49 [1.29 1.71] | 1.72 [1.53 1.92] | 1.69 [1.54 1.85] |
| Manual Social Class | F_2df_=2·80,p=0·061 | 1·03 [0·86 1·24] | 1.13 [0.98 1.29] | 1.30 [1.17 1.45] | 1.13 [1.03 1.24] |
| Rented Accommodation | F_2df_=1·38,p=0·25 | 1·41 [1·14 1·74] | 1.24 [1.04 1.47] | 1.48 [1.30 1.68] | 1.36 [1.22 1.52] |
| Major Financial Problems | F_2df_=0·11,p=0·89 | 1·39 [1·07 1·79] | 1.36 [1.12 1.64] | 1.44 [1.23 1.69] | 1.39 [1.22 1.58] |
| No car access | F_2df_=0·36,p=0·698 | 1·40 [1·01 1·93] | 1.41 [1.08 1.82] | 1.59 [1.29 1.96] | 1.44 [1.20 1.71] |
| Material hardship | F_2df_=0·62,p=0·53 | 1·39 [1·14 1·69] | 1.25 [1.07 1.46] | 1.39 [1.22 1.57] | 1.33 [1.20 1.47] |
| Maternal Education | F_4df_=1·47,p=0·21 >=A level  O-level  CSE/vocational | 1  1·14 [0·91 1·43]  1·19 [0·94 1·50] | 1  1.09 [0.94 1.28]  1.22 [1.03 1.46] | 1  1.30 [1.15 1.47]  1.51 [1.31 1.73] | 1  1.17 [1.05 1.29]  1.27 [1.13 1.43] |

1. Joint F-test for the equality of regression coefficients across the 3 periods and averaged over 100 imputations.

Down-weighting observations for children who are more likely to be followed-up – females and children with more favourable SEP profiles are more likely to be followed.

The imputation model included variables in addition to those included in this analyses that were either associated with missingness or were predictive of depression at age 18. These included maternal age and family adversity and socio-demographics in pregnancy and early childhood. Parameter estimates were averaged over 100 imputed/completed datasets using Rubin rules.^1^ Wald-type tests were used following imputation to compute time invariant/common effect and test time-dependency of the effects of predictors. Sensitivity analyses were conducted after MI to examine departures of the assumption of missingness at random (MAR) in the depression outcome using the weighting approach.^2^

Further sensitivity analyses (available on request) introducing differential dependence of missingness on low SEP suggested that MI estimates can under-estimate risk associated with low SEP under a scenario where missing cases were more likely to be from the more socio-economically deprived groups (and, vice versa, over-estimate the associated risk if missing cases were more likely to be from the more socio-economically privileged groups). However, these departures require rather large dependencies of missingness on the outcome. For instance, they become significant when the probability of depression caseness for those missing the outcome is more than twice that of those observed in the low SEP group exclusively.

A substantial proportion of the participants had missing depression data. For this reason we conducted sensitivity analyses to assess the direction of bias under different scenarios exemplifying departures from the assumption of missing at random (MAR) (shown in Table 3). The results suggest that the missing at random (MAR) estimates tend to contain the not missing at random (NMAR) estimates in their 95% CIs and multiple imputation (MI) estimates are robust under non-differential non-response, even when we grossly under or over-estimate caseness and when departure from MAR is the same between SEP groups.

References

1. Little RJA, Rubin DB. *Statistical Analysis With Missing Data* (2^nd^ Ed.) New York: Wiley, 2002.
2. Carpenter JR, Kenward M, White IR. Sensitivity analysis after multiple imputation under missing at random: a weighting approach. *Stat Methods Med Res* 2007;**16**: 259–275.

Table S3. Univariable relative risk ratios^[[1]](#footnote-1)^ for the association between antenatal SEP and onset of depressive symptoms at different developmental periods defined by age and pubertal status

|  | **Age at first onset** | | | | **Pubertal status at first onset** | | | |
| --- | --- | --- | --- | --- | --- | --- | --- | --- |
| SEP indicator | n | 10 – 12 years | 12 – 16 years | 16 – 20 years | n | Pre-pubertal (stage I) | Pubertal  (stage II-IV) | Post-pubertal  (stage V) |
| Manual social class^3^ | 2545 | 2.04 (1.60, 2.61) | 1.44 (1.16, 1.79) | 1.28 (1.03, 1.59) | 2314 | 1.74 (1.24, 2.44) | 1.48 (1.21, 1.80) | 1.33 (1.04, 1.70) |
|  | Weighted analysis^2^ | 2.57 (1.96, 3.36) | 1.57 (1.25, 1.98) | 1.47 (1.12, 1.91) |  | 2.42 (1.66, 3.54) | 1.66 (1.33, 2.07) | 1.52 (1.12, 2.05) |
| Rented accommodation | 2629 | 3.62 (2.64, 4.96) | 2.08 (1.53, 2.83) | 1.54 (1.11, 2.14) | 2389 | 3.27 (2.15, 4.97) | 2.00 (1.49, 2.67) | 1.98 (1.41, 2.78) |
|  | Weighted analysis^2^ | 4.31 (3.08, 6.05) | 2.08 (1.51, 2.88) | 1.77 (1.19, 2.61) |  | 4.02 (2.54, 6.38) | 2.29 (1.66, 3.15) | 1.90 (1.27, 2.84) |
| Major financial problems | 2481 | 2.46 (1.69, 3.58) | 2.35 (1.67, 3.32) | 1.90 (1.33, 2.72) | 2255 | 2.20 (1.32, 3.65) | 2.47 (1.79, 3.41) | 2.04 (1.39, 3.00) |
|  | Weighted analysis^2^ | 2.65 (1.76, 3.98) | 2.09 (1.46, 2.99) | 2.27 (1.44, 3.44) |  | 2.37 (1.34, 4.17) | 2.42 (1.72, 3.43) | 2.32 (1.43, 3.77) |
| No car access | 2631 | 2.98 (1.79, 4.97) | 1.93 (1.17, 3.19) | 1.11 (0.62, 1.98) | 2390 | 2.74 (1.39, 5.38) | 1.99 (1.24, 3.21) | 0.93 (0.47, 1.83) |
|  | Weighted analysis^2^ | 4.20 (2.44, 7.21) | 2.15 (1.26, 3.67) | 1.19 (0.59, 2.39) |  | 3.51 (1.67, 7.38) | 2.47 (1.49, 4.09) | 1.19 (0.50, 2.86) |
| Material hardship | 2578 | 3.13 (2.37, 4.15) | 2.07 (1.59, 2.69) | 1.50 (1.14, 1.97) | 2343 | 2.99 (2.06, 4.33) | 2.17 (1.70, 2.77) | 1.58 (1.17, 2.14) |
|  | Weighted analysis^2^ | 3.84 (2.84, 5.20) | 2.37 (1.80, 3.13) | 1.62 (1.16, 2.27) |  | 3.76 (2.49, 5.67) | 2.36 (1.81, 3.07) | 1.70 (1.18, 2.44) |
| Low maternal education | 2636 | 2.17 (1.66, 2.85)^4^ | 1.55 (1.24, 1.94) | 1.45 (1.16, 1.81) | 2399 | 1.58 (1.09, 2.29) | 1.57 (1.27, 1.93) | 1.56 (1.22, 2.01) |
|  |  | 3.69 (2.69, 5.05)^4^ | 1.91 (1.44, 2.55) | 1.47 (1.09, 1.99) |  | 2.45 (1.60, 3.77) | 1.93 (1.48, 2.53) | 1.64 (1.18, 2.28) |
|  | Weighted analysis^2^ | 2.58 (1.91, 3.48) ^4^ | 1.78 (1.40, 2.26) | 1.52 (1.16, 1.99) |  | 2.05 (1.35, 3.12) | 1.61 (1.34, 2.11) | 1.68 (1.24, 2.28) |
|  | Weighted analysis^2^ | 4.71 (3.34, 6.64) ^5^ | 2.51 (1.85, 3.40) | 1.88 (1.30, 2.72) |  | 3.15 (1.96, 5.08) | 2.53 (1.89, 3.39) | 1.88 (1.25, 2.83) |

(1) Risk is measured as the risk of the outcome relative to the base outcome (i.e. not experiencing an event/depressive symptoms score >=11) at any age for the duration of follow-up. The sample size in this analysis is restricted in those with complete follow-up.

(2) Manual social class III-V (reference group= non-manual social class I-II)

(3) Weighted analyses use weights based on the inverse of the probability of individuals being selected (i.e. not excluded from the analyses). The measures of risk from the multinomial logit are relative risk ratios (RRR) e.g. the odds favouring experiencing an event in childhood over never experiencing symptoms until 20 years are 2 (weighted 3) times higher for children from families in manual occupations than from non-manual. This analysis is weighted with weights proportional to probability of early drop-out/accounting for the dependence of censoring with duration of follow-up, relaxing the assumption of independence of censoring with time.

(4) O level

(5) CSE/vocational (reference group= high maternal education: A level and above)

The comparison groups in the multinomial model had significant differences in follow-up. A significantly higher proportion (31%) of children who experienced onset of depressive symptoms in the first period (10–12 years) dropped out before 16 years compared with drop-out proportions before 16 years of 16% and 5% for the children who experienced onset of depressive symptoms in the other two periods (12–16 years and 16–20 years) respectively. In other words, depressive symptom onset groups are becoming increasingly similar to the comparison group in terms of follow-up, the later they experienced onset. This could lead to exaggerated impressions of time dependent effects.

This analysis excluded a significant proportion of the study sample, including selectively only those with adequate follow-up in order to definitively assess symptom onset by not allowing observation censoring. As a result, comparing the counts of children with any symptoms according to whether they were included or excluded in this analysis shows a systematic oversampling of cases and under-sampling of the reference group, which reduces with time (onset-period). For these reasons, we also conducted a simple sensitivity analyses by repeating the same analyses but re-weighting the observations of the included children. These weights are inversely proportional to their probability of selection. The selection probabilities were estimated as a function of follow-up duration and any depressive symptoms experienced.

| Table S4: Distribution of gender and SEP indicators for the whole study sample (N=9193) compared to the original cohort (N=14,689) | | | | | | |
| --- | --- | --- | --- | --- | --- | --- |
|  |  | **Study sample**  **N=9193** | | **Original cohort**  **N=14,689** | | |
| Characteristic | Units | No observed | | N | N (%)* | N Missing |
| Female | Binary | 9193 | 4768 (51.9%) | 14689 | 7148 (48.7%) | - |
| Social Class  (manual) | Binary | 7859 | 3183 (40.5%) | 11501 | 5162 (44.9%) | 3188 |
| Rented Accommodation | Binary | 8369 | 1569 (18.8%) | 13025 | 3466 (26.6%) | 1664 |
| Major Financial Problems | Binary | 7785 | 918 (11.8%) | 11904 | 1617 (13.6%) | 2785 |
| No car access | Binary | 8365 | 514 (6.1%) | 13034 | 1406 (10.8%) | 1655 |
| Material hardship | Binary | 8055 | 1864 (23.1%) | 12086 | 3139 (25.6%) | 2603 |
| Maternal Education | Categorical^†^  >=A level  O-level  CSE/vocational | 8271 | 3397 (41.1%)  2916 (35.3%)  1958 (23.7%) | 12416 | 4392 (35.4%)  4296 (34.6%)  3728 (30.0%) | 2273 |
| ^†:^ *Reference Category:A-level and above*  **: % among those observed* | | | | | | |

1. [↑](#footnote-ref-1)
